# Biomonitoring of Human Exposure to Prestige Oil: Effects on DNA and Endocrine Parameters

**DOI:** 10.4137/EHI.S954

**Published:** 2008-10-31

**Authors:** Beatriz Pérez-Cadahía, Josefina Méndez, Eduardo Pásaro, Anunciación Lafuente, Teresa Cabaleiro, Blanca Laffon

**Affiliations:** 1 Toxicology Unit, Dept. Psychobiology, University of A Coruña, Edificio de Servicios Centrales de Investigación, Campus Elviña s/n, 15071-A Coruña, Spain; 2 Dept. Cell and Molecular Biology, University of A Coruña, A Coruña, Spain; 3 Toxicology Laboratory, University of Vigo, Campus Ourense, Ourense, Spain

**Keywords:** Prestige oil, comet assay, prolactin, cortisol, metabolic polymorphisms

## Abstract

Since 1960, about 400 tankers spilled more than 377765 tons of oil, with the *Prestige* accident (Galician coast, NW Spain, November 2002) the most recent. Taking into account the consistent large number of individuals exposed to oil that exists all over the world, it seems surprising the absence in the literature of studies focused on the chronic effects of this exposure on human health. In this work we evaluated the level of DNA damage by means of comet assay, and the potential endocrine alterations (prolactin and cortisol) caused by Prestige oil exposure in a population of 180 individuals, classified in 3 groups according to the tasks performed, and 60 controls. Heavy metals in blood were determined as exposure biomarkers, obtaining significant increases of aluminum, nickel and lead in the exposed groups as compared to controls. Higher levels of genetic damage and endocrine alterations were also observed in the exposed population. DNA damage levels were influenced by age, sex, and the use of protective clothes, and prolactin concentrations by the last two factors. Surprisingly, the use of mask did not seem to protect individuals from genetic or endocrine alterations. Moreover, polymorphisms in genes encoding for the main enzymes involved in the metabolism of oil components were analyzed as susceptibility biomarkers. CYP1A1-3′UTR and EPHX1 codons 113 and 139 variant alleles were related to higher damage levels, while lower DNA damage was observed in GSTM1 and GSTT1 null individuals.

## Introduction

Taking into account the large number of oil spills occurred all around the world, it seems surprising the absence in the international literature of consistent studies dealing with the possible long term effects that oil exposure can cause in humans. Since 1960, 410 tankers spilled more than 377765 tons of oil (Vieites et al. 2004), with the Prestige accident (Galician coast, NW Spain, November 2002) the most recent. Consequently, a considerable number of individuals experienced an oil exposure all over the world in different ways and for different periods of time. Furthermore, due to the social commotion unleashed by these events and the time pressure under which the reaction to the spills takes place, the measures adopted in terms of tasks organization, implementation of protective devices for individuals and establishment of the work shifts are frequently not adequate. Nevertheless, although some works evaluated the effects of oil spills on human health, all of them were focused on acute and/or psychological endpoints (Palinkas et al. 1993; Morita et al. 1999). This fact stands out when considering the genotoxic and endocrine disruptor properties of the three main groups of substances that European Communitarian Normative establishes as to be taken into account when examining the potential risk caused by the oil: volatile organic compounds (VOC), polycyclic aromatic hydrocarbons (PAH) and heavy metals (Commission Directive 93/67/EEC, Commission Regulation (EC) No. 1488/94). The International Agency for Research on Cancer (IARC) classified fuel oils No. 6, such as Prestige oil, as possible human carcinogens (Group 2B).

A big human mobilization took place in Galicia after *Prestige* oil spill in order to try to alleviate the catastrophic consequences caused in the environment. This fact positioned these people as integrants of an exposed population probably affected by the oil. The Spanish National Research Council determined the composition of Prestige oil as 50% aromatic hydrocarbons, 22% saturated hydrocarbons and 28% resins and asphalthene (CSIC 2003a). The aromatic hydrocarbon fraction included light aromatic hydrocarbons (naphthalene and its alkylated derivatives), high molecular weight hydrocarbons (complex mixtures of asphalthenes and PAH) and volatile aromatic hydrocarbons (primarily benzene, toluene and xylenes), although the latter were in low proportion in the original oil. Moreover, it contained six PAH categorized by the IARC as probable or possible human carcinogens (benzo[a]anthracene, benzo[b]fluoranthene, benzo[k]fluoranthene, benzo[a]pyrene, dibenz[ah]anthracene and indene[1,2,3-cd]pyrene), and included in the 16 PAH designated by the United States Environmental Protection Agency (USEPA) as primary contaminants. A detailed chemical analysis of PAH contained in the *Prestige* oil that reached the coast was given in Pérez-Cadahía et al. (2004). Furthermore, data on presence of different quantities of heavy metals in emulsified samples of *Prestige* oil were previously reported (CSIC 2003b; Albaigès et al. 2006). Under these circumstances, it seemed necessary to design and to develop a study focused on the consequences of the exposure, considering the main toxicological properties of oil integrants. Therefore we performed an initial study comprising a pilot population (68 exposed vs. 42 controls), and results obtained suggested the existence of certain genotoxicity and endocrine alterations in the exposed individuals (Pérez-Cadahía et al. 2006 and 2007).

In view of these results, we decided to enlarge the study with the aim of checking the validity of these previous data. Exposed individuals were classified in three groups: volunteers (V) exposed only for 5 days, and hired staff who worked either manually for 4 months (MW) or using high-pressure cleaning machines for 3 months (HPW). Comet assay was employed as an indicator of the genotoxic effects, and plasmatic levels of prolactin and cortisol were determined to evaluate the potential alterations in the endocrine status. Complementarily, several genetic polymorphisms in the enzymes involved in the metabolism of VOC and PAH contained in the oil (CYP1A1-3′UTR, EPHX1 codons 113 and 139, GSTP1 codon 105, and GSTM1 and GSTT1 deletion polymorphisms) were analyzed in order to evaluate their influence on DNA damage levels and endocrine toxicity.

## Material and Methods

### Study population

Three different groups of exposed individuals were examined, 61 volunteers (V) involved in the manual collection of oil in March 2003 during 5 days for 4 h/day, 59 hired workers (MW) that collected oil from December 2002 to April 2003 for 6.5 h/day, and 60 hired workers who employed high-pressure machines (HPW) to clean rocks from February to May 2003 for 6.5 h/day. In the exposed population 38.3% were males, 37.8% smokers (8.81 ± 10.99 packs-year), and the mean age was 32.26 ±11.68 years. Protective devices used included clothes (waterproof overalls) and cellulose mask, although some individuals did not wear them (18.3% for clothes and 6.7% for mask). Sixty non-exposed individuals were included as control group; 51.1% were males, 31.7% were smokers (3.92 ±4.64 packs-year), and the mean age was 23.31 ± 4.89 years. An informed consent was signed and a detailed questionnaire on lifestyle and consumption habits was filled in by each individual included in the study. This study was performed in accordance with the ethical standards laid down in the 1964 Declaration of Helsinki.

### Sample collection

Samples were collected between March and May 2003. Peripheral blood was drawn early in the morning before the working shift by venipuncture in three types of tubes, one containing lithium heparine for determining blood concentrations of heavy metals, EDTA containers for DNA extraction and genotyping, and special BD Vacutainer^™^ CPT^™^ for leukocyte isolation for the comet assay. All samples were codified and refrigerated until arrival to the laboratory, where they were processed according to each established protocol.

### Heavy metals in blood

In order to determine heavy metal intake, blood concentrations of aluminum, cadmium, nickel, lead and zinc were analyzed by graphite furnace atomic absorption spectrophotometry (GFAAS) after microwave digestion (Pérez-Cadahía et al. 2007). Accuracy of the methodology was obtained by calibration against aqueous standards (RSD ≤ 5%). The lowest level of sensitivity was 0.02 μg/l. Samples of the whole experiment were analyzed within the same assay to avoid interassay variations, and the intraassay coefficient of variation was 4%.

### Comet assay

Mononuclear leukocyte isolation was made using BD Vacutainer™ CPT™ Cell Preparation Tubes (Becton Dickinson) following manufacturer’s instructions. Cells were resuspended in freezing medium (40% RPMI 1640, 50% fetal bovine serum and 10% DMSO) and stored at −80 ºC in a Nalgene® Cryo 1 ºC Freezing Container (Nalgene Nunc International) until their use.

Leukocytes were thawed at 37 ºC and their viability was checked with trypan blue, being always ≥85%. Alkaline comet assay was performed basically as described by Singh et al. (1988) with minor modifications (Laffon et al. 2002). One hundred cells per donor (50 per replicate slide) were analyzed by a blind scorer employing the QWIN Comet software (Leica Imaging Systems, Cambridge, U.K.). The percentage of DNA in the comet tail (%TDNA) was used as DNA damage parameter.

### Prolactin and cortisol in plasma

Specific radioimmunoassays (RIA) were used to determine plasmatic prolactin and cortisol levels using two commercial kits, previously tested: Prolactin IRMA DSL-4500 and Cortisol RIA DSL-2000 (Diagnostic Systems Laboratories, Webster, TX, U.S.A) following manufacturer’s recommendations. The intraassay coefficients of variation were 7.4% and 4.3% for prolactin and cortisol, respectively. All samples were measured in the same assay to avoid interassay variations. Sensitivities of the assays were 0.1 ng/ml for prolactin and 0.11 μg/dl for cortisol.

### Genotype analysis

DNA was extracted from 300 μl of whole peripheral blood using Puregene™ DNA isolation kit (Gentra Systems, Minneapolis, U.S.A.). Genetic polymorphisms consisting in single nucleotide changes were evaluated by means of PCR-RFLP techniques following Laffon et al. (2003a) for CYP1A1-3′UTR and EPHX1 codon 113, Laffon et al. (2006a) for EPHX1 codon 139 and Saarikoski et al. (1998) for GSTP1 codon 105. GSTM1 and GSTT1 deletion polymorphisms were analyzed employing a multiplex PCR method (Laffon et al. 2003b) in which a fragment of the β-globin gene is simultaneously amplified as an internal control to verify the proper functioning of the PCR reaction. All genotype analyses were performed at least in duplicate to confirm the study results.

### Statistical analysis

Analysis of Variance (ANOVA) and Tukey’s test were used to evaluate the existence of differences among groups. The contribution of exposure and potential confounding factors (sex, age, smoking habits, and use of protective devices) to the genotoxic and endocrine response variables considered was assessed by means of multifactorial ANOVA analysis. The associations between two variables were analyzed by Pearson’s correlation. The level of statistical significance was set at 0.05, and all analyses were performed using the SPSS for Windows statistical package, version 14.0 (IL, U.S.A).

## Results

### Exposure biomarkers

**Figure 1.**
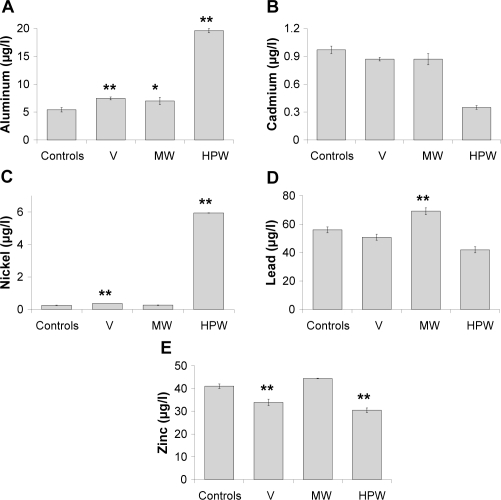
Heavy metal blood concentrations (mean ± SE) in the studied populations: aluminum **A**), cadmium **B**), nickel **C**), lead **D**) and zinc **E**). *P < 0.05; **P < 0.01, significant difference with regard to controls. Results adjusted by sex, age and tobacco smoking.

Exposure levels were measured by determining blood concentrations of aluminum, cadmium, nickel, lead and zinc. Results obtained adjusted by sex, age and smoking habits are gathered in Figure 1. Statistically significant increases in aluminum levels were observed in the three exposed groups regarding to controls. Cadmium evaluation did not reveal significant variations among groups. Nickel blood concentrations experienced increases in the three exposed populations; statistical significance was reached in V and HPW groups although the difference was more obvious in the last one. Regarding to lead, higher levels of this metal were detected in the individuals belonging to MW group. Finally, zinc concentrations significantly decreased both in V and HPW groups. Associations between heavy metals concentrations and the effect biomarkers evaluated were analyzed, but only a significant correlation between cadmium and cortisol levels (r = 0.204, *P* < 0.01) was obtained.

### Effect biomarkers

**Figure 2.**
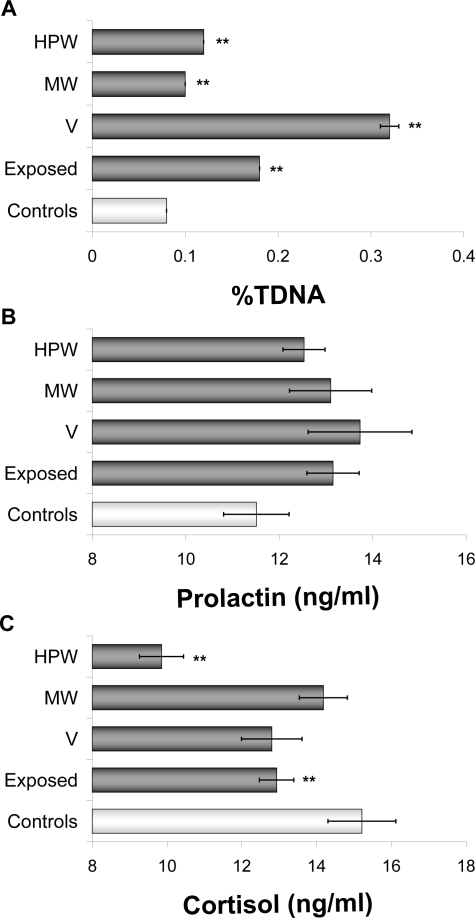
Effect of exposure on DNA damage expressed as %TDNA **A**), prolactin **B**) and cortisol **C**) plasmatic levels (mean ± SE). **P < 0.01, significant difference with regard to controls. Results adjusted by sex, age and smoking habits.

Figure 2 shows the results of comet assay and endocrine determinations in the different exposure groups, after adjusting by sex, age and smoking habits. All exposed individuals showed significantly increased DNA damage levels. Specially, V group experienced the most noticeable variation followed by HPW. Prolactin plasmatic concentrations were higher in the three exposed groups than in the controls, although the differences were never enough to reach statistical significance. On the contrary, cortisol concentrations decreased in all exposed groups; the difference was significant in the total exposed population and in HPW.

The contribution of exposure and potential confounding factors to the genotoxic and endocrine response variables considered was assessed by means of multifactorial ANOVA analysis.

The analysis of the influence of sex, age and smoking habits on DNA damage and endocrine parameters is summarized in Table 1. When considering these factors individually after adjusting by exposure in the multifactorial ANOVA analysis, comet assay results were influenced by sex and age (significant *P* values); both of them remained significant when they were mutually adjusted, i.e. introduced together in the same model (partial *P* values 0.003 and 0.0001, respectively, model *P* value < 0.001). Furthermore, there was a significant correlation between %TDNA and age (Pearson coefficient r = 0.043, *P* < 0.01). Significant effect of sex on prolactin concentrations (*P* = 0.017) was also obtained, but no effect was observed on cortisol levels. Although smoking did not influence any response variable evaluated, smokers presented DNA damage levels significantly higher than non-smokers in V group (0.375 ± 0.012 *vs*. 0.304 ± 0.007, *P* < 0.01) but significantly lower in MW group (0.087 ± 0.003 vs. 0.111 ±0.003, *P* < 0.01). With regard to the effect of wearing protective devices during cleaning labors, significantly increased DNA damage and prolactin levels (*P* = 0.003 and 0.049, respectively) were associated with the absence of using clothes, while no influence of wearing mask on any parameter was observed.

### Susceptibility biomarkers

We analyzed the possible influence of some important polymorphisms in genes involved in the biotransformation pathways of oil components on the DNA damage and hormone levels. After confirming that CYP1A1, EPHX1 and GSTP1 studied polymorphisms were in Hardy-Weinberg equilibrium, individuals were grouped depending on the most suitable heredity model for each gene according to the results (Analysis of Variance), since no data were available in the literature, excepting for EPHX1 (Vesell, 1979).

Table 2 shows the influence of the studied polymorphisms on the genetic damage levels. Significant increases in % TDNA in control and V individuals carriers of the CYP1A1^*2A^ variant allele were obtained. With respect to the effect of EPHX1 polymorphisms, results displayed a statistically significant increase in DNA damage related to the presence of the codon 113 variant allele in individuals from control, MW and HPW groups; nevertheless for V the effect was the opposite. Data from the analysis of EPHX1 codon 139 polymorphism showed a decrease of %TDNA in control individuals carrying the variant allele, while in V and MW this value was increased. Presence of GSTP1 codon 105 variant allele was associated with a decrease of the strand breaks level in V group, exerting the inverse effect in total exposed population and HPW. GSTM1 null individuals generally showed decreased levels of DNA damage with regard to the positive ones, reaching statistical significance in total exposed and MW groups. Similarly, GSTT1 null MW and HPW individuals presented significantly lower rates of DNA strand breaks than positive subjects.

Genetic polymorphisms analyzed seemed not to significantly influence plasmatic hormone levels, excepting for decreases in prolactin concentrations both in controls and exposed carrying the CYP1A1^*2A^ allele, and increases in cortisol levels in control individuals carrying the variant GSTP1-105^Val^ allele and in GSTT1 null V subjects.

## Discussion

Due to the intense maritime traffic of hazardous cargo, there is a considerably large human population exposed worldwide to the effects of diverse toxic agents. Oil spills are perhaps the most frequent and serious disasters related to this topic, representing an important problem of Public Health. In November 2002, the wreck of the Prestige tanker caused the enormous spill of about 63,000 tons of oil that reached Galician coast as three black tides. The research activity after the *Prestige* oil spill was almost exclusively related to the environmental effects on the flora and fauna, the scarce works dealing with human health were just set in the framework of acute effects of the exposure (Suárez et al. 2005). Nevertheless, taking into account that the main damaging properties of oil integrants are related to genotoxic and/or carcinogenic processes, the evaluation of chronic effects of oil exposure deserves special relevance, further considering the absence of studies on this topic in the international literature and the big amount of oil spills happened all over the world in the last decades.

In our study, we evaluated blood concentrations of several heavy metals as exposure bio-markers. Heavy metals analyzed are harmful agents contained in *Prestige* oil and they are chemically very stable, i.e. not easily modified, and not subjected to environmental degradation or metabolic transformation. Therefore they give a constant and comparable measure of the exposure levels. Toxicity of heavy metals derived both from their carcinogenic character (Hayes, 1997) and from their interaction with other organic systems as immunologic or endocrine (Lafuente et al. 2005) has been widely reported in the literature. Among all heavy metals contained in the Prestige oil aluminum, cadmium, nickel and lead were selected to be analyzed in this study because of their recognized carcinogenicity (IARC groups 1 and 2B), and zinc for having some opposite effects to the other metals, as it is detailed below. General increases in the exposed groups compared to controls were observed for all metals, excepting for zinc that showed the opposite behavior. This fact could be related to the increase of other toxic metals, due to interferences in the absorption process or to the protective role played by this metal, as it is an essential constituent of very important structures such as the zinc fingers (Waalkes, 2003). Lutzen et al. (2004) reported that incubation of human cells in a zinc excess prior to their treatment with cadmium reverts the suppressive effect of this metal on the mismatch repair system. Although exposure to Prestige oil involved an increase in blood concentration of heavy metals, aluminum and nickel being good indicators of this exposure, none of the obtained values reached either the biological limit values adopted by the Spanish National Institute of Occupational Safety and Hygiene (INSHT 2007) or the toxic biological levels proposed by Repetto and Repetto (2005). On the other hand, VOC concentrations in the environment where these exposed groups had been working were determined in our previous study, obtaining significantly higher levels in V group than in MW and HPW groups (Pérez-Cadahía et al. 2007).

Several studies described the genotoxic character of oil integrants separately, but there are no reports in the literature evaluating the effect of this complex mixture as a whole. Comet assay detects DNA primary lesions, mainly single and/or double strand breaks, as well as incomplete excision repair processes. So it gives a good estimate of a very early response of the organism to the effects of genotoxic agents on DNA integrity. This is a key point in the interpretation of the results provided by this assay, as these lesions are usually quite fast repaired and thus the damage reflected is relatively recent. Furthermore, its high sensitivity provides this assay with a high potential for quantifying DNA damage in populations exposed to low levels of genotoxic agents (Sul et al. 2003). In this work, an increase in DNA damage was detected in all exposed groups related to controls, especially in V, agreeing with environmental VOC concentrations previously found. In this regard, several recent studies suggest the possible existence of an adaptive response to stress situations (Gourabi and Mozdarani, 1998; Bonassi et al. 2003). The adaptive response means that individuals subjected to a prolonged low or medium intensity exposure to an agent, would exhibit a lower response to a subsequent exposure than never exposed individuals. Taking into account that comet assay detects with high sensitivity an immediate response of the organism to the exposure, the adaptive response seems a suitable explanation for the higher damage values obtained in V group regarding to MW and HPW, who had been exposed to oil for 4 and 3 months, respectively, prior to the sample collection.

Non-significant increases in prolactin concentrations were obtained in all exposed groups regarding to controls. Lead stands out among heavy metals contained in the oil by its well-documented endocrine disruptor properties. It decreases the levels of dopamine that controls prolactin secretion by the adenohypophysis by means of an inhibitory mechanism (Roses, 2001). According to this, an exposure to lead as the one reflected by blood levels of Prestige oil exposed individuals was expected to increase prolactin plasmatic concentration. On the contrary cortisol levels in exposed individuals decreased with regard to controls, confirming results obtained in our previous work (Pérez-Cadahía et al. 2007).

Sex is a very influential factor on cytogenetic genotoxicity tests (Lazutka et al. 1994; Bonassi et al. 2001), but its effect on comet assay is much less clear, since several authors reported significant differences between genders like in this study (Bajpayee et al. 2002) or no differences at all (Zhu et al. 2000). As expected, prolactin levels were increased in females from all populations, but much higher increase was observed in the exposed (11.83 ± 0.71 vs. 14.69 ± 0.04, *P* < 0.05) than in the controls (10.96 ± 0.96 vs. 11.86 ± 1.02), pointing to a sex influence related to the exposure that could determine a higher susceptibility of females to the effects of oil exposure on prolactin secretion. On the other hand, no effect of sex on cortisol concentrations was observed.

Our results showed a direct relationship between age and %TDNA, agreeing with the fact that age has been widely related to increases in the background damage levels, losses in the efficacy of the repair mechanisms and the subsequent damage accumulation. The decrease of the defense mechanisms against toxicity related to age was found to be associated with the generation of reactive oxygen species and the decrease of agents participating in the detoxification processes as glutathione (Rihs et al. 2005). Studies dealing with this factor report varied conclusions; in some cases no influence was detected by means of the comet assay (Sul et al. 2003) while other authors described associations between genetic damage levels and age (Lam et al. 2002). On the contrary, influence of age on endocrine parameters evaluated was not obtained.

Tobacco smoke is especially associated with the exposure evaluated in this work due to the great number of highly toxic component substances that share with oil (e.g. benzo[a]pyrene, benzene, cadmium, etc.). Tobacco consumption did not influence comet assay results; however smoking was related to increase in genetic damage in V group but decrease in MW chronically exposed group. Garaj-Vrhovac and Kopjar (2005) suggested that enhanced DNA repair mechanisms in smokers might be the reason why the effect of an additional occupational exposure was more pronounced in non-smokers. Faust et al. (2004) also supported this hypothesis proposing that the stimulation of DNA repair or detoxifying mechanisms induced by xenobiotics coming from occupational exposures are important in the attenuation of the effect of chemical agents from the tobacco smoke, and this could happen also in the inverse way.

Protective devices used by the exposed individuals included waterproof clothes and cellulose mask, aimed at avoiding dermal and respiratory exposure, respectively. Clothes seemed to protect against increase in DNA damage and plasmatic prolactin levels, but surprisingly wearing mask did not influence genetic or endocrine parameters. Thus, it seems that inhalant exposure risk would seem to have been minimal compared to dermal. In contrast, use of mask determined a significant decrease in DNA damage levels (comet assay) in individuals engaged in the cleaning and autopsies of Prestige oil contaminated birds (Laffon et al. 2006b). The inappropriate characteristics of the devices employed or their inadequate utilization by the individuals are probably the reasons that underlie these unexpected results.

Nowadays, the existence of polymorphisms in several genes involved in metabolic pathways are widely described and considered key in epidemiological studies due to their repercussions on the activation/detoxification processes and consequently on damage induction. Therefore an important part of this work dealt with the analysis of the influence of the main metabolic polymorphic variants on the effect biomarkers evaluated.

Cytochrome P450 (CYP) enzymes are considered the first defense against lipophilic compounds. Specifically, CYP1A1 catalyzes the formation of mutagenic intermediaries of many PAH (Savas et al. 1997). One of the most studied polymorphisms in this gene is located in the 3′-untranslated region (UTR) (*1A/*2A). Data coming from comet assay showed statistically significant increases in DNA damage levels associated with the presence of the variant allele in individuals from the control and V groups, agreeing with the higher inducibility of this enzyme (Redon et al. 2006), and the consequent increased production of highly genotoxic intermediates.

Two EPHX1 polymorphisms affecting the activity of the resulting microsomal epoxide-hydrolase enzyme and established as risk factors against several exposures were studied. Codon 113 polymorphism was reported to decrease enzyme activity (Hasset et al. 1994) and results obtained showed increases in DNA damage in controls, MW and HPW carrying the variant allele, in agreement with the general detoxifying activity of the enzyme.

EPHX1 codon 139 polymorphism increases epoxide-hydrolase activity (Hasset et al. 1994). This polymorphism is considered a special risk factor in relation to PAH exposures, since dihydrodiols resulting from the action of this enzyme can be transformed again by specific CYPs giving rise to even more reactive species, the dihydrodiolepoxides (Pavanello and Clonfero, 2000). Consequently, the higher activity generated by codon 139 variant is an unfavorable factor in PAH exposures. In fact, epoxide-hydrolase activity is considered part of the PAH activation route (Norppa, 2004). In this work higher % TDNA was detected in individuals from the exposed population carrying the variant allele, according to the higher production of reactive PAH metabolites, and lower in the control population, suggesting an exposure-associated effect.

Although glutathione conjugation mediated by glutathione *S*-transferases (GST) is not a priority pathway in liver, it acquires greater importance in other tissues where epoxide-hydrolase activity is low (Norppa, 2004). It is well known that GSTP1 enzyme takes part in the pulmonary detoxification of inhaled PAH and in oxidative stress processes (Buschini et al. 2003). GSTP1 enzymes with Val105 have a seven-fold higher efficiency for PAH dihydrodiol-epoxides (Hu et al. 1997) but this form is three-fold less effective using low size substrates as 1-chloro-2, 4-dimethylbenzene (Ali-Osman et al. 1997). In this study GSTP1 variant allele was associated with lower genetic damage rates in the total exposed population and V, while HPW showed the opposite tendency, and both differences were statistically significant. This apparent controversy could be related to the mixture of compounds constituting the oil, containing both PAH and low size hydrocarbons as benzene and styrene, or to the differences in the exposure determined by the use of high-pressure machines by HPW individuals.

GSTM1 is involved in benzo[a]pyrene dihydrodiol-epoxides detoxification (Norppa, 2004). Results obtained showed decreases in DNA damage rates associated with the null genotype both in controls and exposed groups. In this regard, Parl (2005) found an increased risk of breast cancer among GSTM1 positive women, and suggested that the combination of all GST conjugation activities may cause glutathione depletion and therefore result counterproductive.

Lastly, GSTT1 null individuals belonging to MW and HPW groups had lower % TDNA than the positive ones. It is well known that gene expression induction or inhibition by external agents is one of the most influential factors on the detoxifying role of this kind of enzymes. An inhibitor effect of benzo[a]pyrene on GSTT1 expression was reported (Kemp et al. 2006) and this could help to explain results obtained in these groups with chronic oil exposure (MW and HPW), although the low number of null GSTT1 individuals must be also taken into account. Nevertheless, the fact that this compound or other PAH is able to alter the expression balance of GST requires further investigation.

Influence of the studied genetic polymorphisms on plasmatic levels of prolactin and cortisol was hardly obtained, suggesting that mechanisms that regulate the secretion of these hormones are not sensitive enough to the small changes in the internal metabolite dose due to the presence of variant alleles in the analyzed genes.

In brief, individuals exposed to Prestige oil presented significant increases in blood aluminum, nickel and lead regarding to controls. Higher levels of genetic damage and endocrine alterations (prolactin and cortisol) were also observed in the exposed population. DNA damage levels were influenced by age, sex, and the use of protective clothes, and prolactin concentrations by the last two factors. Surprisingly, the use of mask did not seem to protect individuals from genetic or endocrine alterations. Regarding to susceptibility biomarkers, CYP1A1-3′URT and EPHX1 codons 113 and 139 variant alleles were related to higher damage levels, while absence of GSTM1 and GSTT1 activities gave rise to lower DNA damage. In view of all the results obtained, it ought to be highlighted the need for developing this kind of studies after oil spills, on one hand to increase the knowledge on the effect of the exposure on human health, as they are more frequent than expected, and on the other hand to define the factors to be taken into account when designing an action protocol before such catastrophes, in order to try to minimize the risk for the individuals.

## Figures and Tables

**Table 1.** Influence of sex, age, smoking habits and the use of protective clothes and mask on DNA damage and endocrine parameters. Models adjusting by exposure. CI: confidence interval, R^2^: correlation coefficient.

**Model**	**Unstandardized coefficients β**	**95% CI**	**Partial *P* value**	**R^2^**	**Model *P* value**
1. Comet assay (%TDNA)					
Males vs. females	0.015	0.009 to 0.022	0.000	0.140	0.000
Age (years)	0.002	0.002 to 0.002	0.000	0.145	0.000
Non-smokers vs. smokers	−0.007	0.013 to 0.000	0.064	0.139	0.000
No clothes vs. clothes^a^	0.020	0.007 to 0.033	0.003	0.119	0.000
No mask vs. mask^a^	0.003	− 0.008 to 0.014	0.621	0.118	0.000
2. Prolactin (ng/ml)					
Males vs. females	−2.386	−4.346 to −0.427	0.017	0.041	0.064
Age (years)	−0.048	−0.170 to 0.074	0.438	0.015	0.533
Non-smokers vs. smokers	−0.722	−2.760 to 1.316	0.486	0.017	0.455
No clothes vs. clothes^a^	4.117	0.022 to 8.212	0.049	0.028	0.226
No mask vs. mask^a^	0.305	−3.907 to 4.516	0.887	0.003	0.928
3. Cortisol (ng/ml)					
Males vs. females	−0.590	−2.215 to 1.036	0.475	0.108	0.000
Age (years)	0.044	−0.056 to 0.144	0.389	0.113	0.000
Non-smokers vs. smokers	−1.074	−2.747 to 0.600	0.207	0.112	0.000
No clothes vs. clothes^a^	−1.597	−4.430 to 1.235	0.267	0.125	0.000
No mask vs. mask^a^	0.858	−1.940 to 3.656	0.546	0.102	0.000

a Models excluding control individuals.

**Table 2.** Influence of metabolic polymorphisms on %TDNA (mean ± SE). N is indicated between brackets.

**Gene**	**Controls**	**Exposed**	**V**	**MW**	**HPW**
CYP1A1^*1A/*1A^	0.079 ± 0.002 (*46*)	0.176 ± 0.003 (*137*)	0.307 ± 0.007 (*48*)	0.104 ± 0.002 (*41*)	0.122 ± 0.003 (*48*)
CYP1A1^*1A/*2A^	0.089 ± 0.004^a^ (*12*)	0.180 ± 0.005 (*35*)	0.378 ± 0.016^a^ (*10*)	0.099 ± 0.004 (*14*)	0.117 ± 0.005 (*11*)
CYP1A1^*2A/*2A^	(*0*)	0.657 ± 0.072 (*1*)	0.657 ± 0.072 (*1*)	(*0*)	(*0*)
EPHX1–113^Tyr/Tyr^	0.075 ± 0.002 (*41*)	0.183 ± 0.003 (*99*)	0.352 ± 0.009 (*35*)	0.102 ± 0.003 (*38*)	0.112 ± 0.003 (*50*)
EPHX1–113^Tyr/His + His/His^	0.086 ± 0.003^a^ (*19*)	0.174 ± 0.003 (*81*)	0.292 ± 0.008^a^ (*26*)	0.103 ± 0.003^a^ (*21*)	0.128 ± 0.003^a^ (*49*)
EPHX1–139^His/His^	0.083 ± 0.0.002 (*44*)	0.179 ±0.003 (*119*)	0.316 ± 0.007 (*44*)	0.098 ± 0.003 (*38*)	0.121 ± 0.003 (*37*)
EPHX1–139^His/Arg + Arg/Arg^	0.067 ± 0.003^a^ (*16*)	0.177 ± 0.004 (*61*)	0.343 ± 0.012^b^ (*17*)	0.110 ± 0.004^a^ (*21*)	0.121 ± 0.004 (*23)*
GSTP1^Ile/Ile^	0.085 ± 0.002 (*30*)	0.176 ± 0.003 (*88*)	0.355 ± 0.010 (*26*)	0.110 ± 0.003 (*29*)	0.109 ± 0.003 (*33*)
GSTP1^Ile/Val + Val/Val^	0.079 ± 0.002 (*25*)	0.180 ± 0.003^a^ (*86*)	0.296 ± 0.008^a^ (*33*)	0.094 ± 0.003 (*26*)	0.138 ± 0.004^a^ (*27*)
GSTM1 positive	0.081 ± 0.002 (*33*)	0.203 ± 0.003 (*94*)	0.329 ± 0.007 (*40*)	0.114 ± 0.003 (*28*)	0.118 ± 0.004 (*26*)
GSTM1 null	0.076 ± 0.002 (*27*)	0.151 ± 0.003^a^ (*86*)	0.312 ± 0.011 (*21*)	0.093 ± 0.003^a^ (*31*)	0.123 ± 0.003 (*34*)
GSTT1 positive	0.078 ± 0.002 (*58*)	0.178 ±0.002 (*161*)	0.329 ± 0.001 (*55*)	0.114 ± 0.001 (*55*)	0.124 ± 0.003 (*51*)
GSTT1 null	0.112 ± 0.010 (*2*)	0.181 ± 0.007 (*19*)	0.355 ± 0.018 (*6*)	0.086 ± 0.007^b^ (*4*)	0.104 ± 0.005^a^ (*9*)

a *P* < 0.01;

b *P* < 0.05, significant difference regarding to the wild type homozygote or positive group.
